# IL6ST: A Novel Therapeutic Target for Managing and Treating Colorectal Cancer Via Ferroptosis

**DOI:** 10.5152/tjg.2024.23353

**Published:** 2024-09-01

**Authors:** Kun Zhao, Baoguo He, Kuijin Xue, Bin Cao, Keyu Ren, Yanchun Jin, Shanwei Rong, Liangzhou Wei, Hongyun Wei

**Affiliations:** Department of Gastroenterology, Affiliated Hospital of Qingdao University, Qingdao, Shandong, China

**Keywords:** IL6ST, ferroptosis, inflammation, colorectal cancer

## Abstract

**Background/Aims:**

Inflammation is an essential driver of colorectal cancer (CRC). Identifying phenotypes and targets associated with inflammation and cancer may be an effective way to treat CRC.

**Materials and Methods:**

R was used to analyze interleukin 6 cytokine family signal transducer (IL6ST) expression in The Cancer Genome Atlas Colon Adenocarcinoma database. Immunohistochemistry, western blotting, and quantitative PCR were used to detect IL6ST and ferroptosis-related genes expression in our cohort. Receiver operating characteristic curves evaluated the specificity and sensitivity of IL6ST to predict CRC. Cell counting kit-8 investigated cell viability. Mitochondrial morphology, total iron, and reactive oxygen species (ROS) levels were evaluated to assess cell ferroptosis. The correlation of IL6ST and immune cells filtration were also analyzed based on R.

**Results:**

IL6ST was significantly upregulated in CRC tissues (*P* < .05). The specificity and sensitivity of IL6ST for predicting CRC were high (area under the curve (AUC): 0.919, CI: 0.896-0.942). IL6ST was significantly associated with ferroptosis-related genes. IL6ST knockdown decreased SW480 cells viability (knockdown vs. vector, *P* = .004), promoted the ferroptosis phenotype, and increased iron accumulation (knockdown vs. vector *P* = .014) and ROS production (knockdown vs. vector P = .005). IL6ST upregulation increased SW620 cells viability (overexpression vs. blank, *P* = .001), inhibited the ferroptosis phenotype, and decreased iron accumulation (overexpression vs. vector *P* = 0.006) and ROS production (overexpression vs. vector *P* = .05). IL6ST increased FTH1 and GPX4 expression and reduced PTGS2, NOX1, and ACSL4 expression (*P* < .01). Additionally, IL6ST level is linked to immune cell infiltration. A higher enrichment score of T cells was observed in IL6ST up-regulated group.

**Conclusion:**

IL6ST inhibits ferroptosis and may be a potential novel therapeutic target in CRC via the modulation of ferroptosis.

**Main Points**

- Inflammation is an essential driver of colorectal cancer (CRC).
- Identifying phenotypes and targets associated with inflammation-related cancer may be an effective method of CRC treatment.
- Interleukin 6 cytokine family signal transducer, an inflammatory cytokine, inhibits ferroptosis via protecting mitochondrial integrity and decreasing iron accumulation and reactive oxygen species production.

## Introduction

Colorectal cancer (CRC) is one of the most prevalent cancers and a major cause of death globally.^1,2^ Chemotherapy is often recommended for patients in advanced stages as a first-line treatment.^3,4^ However, these first-line options are not cost-effective and do not demonstrate long-term overall survival.^4-8^ Furthermore, despite the emergence of immunotherapy for CRC, offering hope,^9^ only a minority of patients with CRC demonstrate significant responses to these agents.^9,10^ Treatment non-response and CRC progression are important causes of incurable disease and increased mortality. Accordingly, identifying new therapeutic targets for CRC during the early stages is necessary. Inflammation is suggested to be an important driver of CRC cancer.^11^ Identifying the phenotypes and markers associated with inflammation-associated cancer may be an effective way to manage and treat CRC.

Ferroptosis is a regulated form of cell death characterized by iron aggregation, lipid peroxidation, and glutathione (GSH) depletion, which can affect inflammation by regulating the number of immune cells and their function or triggering an inflammatory response.^12,13^ Evidence from published studies indicates that ferroptosis is involved in inflammation-related intestinal disease, such as ulcerative colitis and Crohn’s disease.^14^ Excess iron administration leads to excessive iron in the intestine, leading to an imbalance in reactive oxygen species (ROS) generation and disturbance of the intestinal microbiota, which can aggravate inflammatory bowel disease (IBD).^15^

Moreover, ferroptosis has been reported to be involved in CRC development.^16^ For instance, ferroptosis-related genes can effectively predict CRC prognosis.^17,18^ Colorectal cancer patients with low ferroptosis scores showed better progression-free survival and chemotherapy response.^19^ Tagitinin C induced ferroptosis in CRC cell-lines and inhibits CRC cell growth.^20^ Ginsenoside Rh4 has been reported to inhibit CRC proliferation by inducing ferroptosis.^21^ Cetuximab, an approved treatment for metastatic CRC, promotes ferroptosis by regulating the p38/Nrf2/HO-1 axis in KRAS mutant CRC.^22^ These drugs are new and potentially effective against CRC. However, drugs associated with ferroptosis do not target molecules in the early stages of CRC. Finding combined early stage therapeutic targets with ferroptosis-inducing agents will be beneficial in the treatment of early stages of CRC.

Interleukin 6 cytokine family signal transducer (IL6ST) is a protein that serves as a co-receptor for interleukin-6 (IL-6), interleukin-11 (IL-11), and related cytokines. These cytokines activate the JAK–STAT3 and PI3K–AKT–mTORC1 pathways via IL6ST.^23^ Interleukin-6 and IL-11 have been reported to be associated with STAT3 activation in inflammation-related gastrointestinal cancers.^24^ STAT3 is one of the main pathways that links inflammation to cell proliferation and cancer.^25,26^ In addition, IL6ST links colon inflammation to colon epithelial regeneration by triggering YAP and Notch signaling pathways.^27^

Interleukin 6 cytokine family signal transducer activation in *Adenomatous polyposis coli (APC)* loss mice can accelerate CRC development depending on SFK and JAK pathways.^28^ Whether IL6ST is related to ferroptosis in CRC is unknown, and the current study intends to provide preliminary evidence for future clinical translation by observing whether IL6ST affects ferroptosis. This study offers a potential target for the combination of ferroptosis inducers in the early treatment of CRC.

## Materials and Methods

### Sample Collection and Immunohistochemistry

Colorectal cancer tissues and non-tumor adjacent tissues were collected from patients who underwent surgery at the Affiliated Hospital of Qingdao University. Ten CRC tissues and 10 non-tumor adjacent tissues from CRC patients were included from 2021 to 2022 for further immunohistochemical (IHC) analysis. This study was approved by the Ethics Committee of the Affiliated Hospital of Qingdao Universtiy (approval number: QYFYWZLL26573, date: May 10, 2021), and informed consent was obtained from all the participants. For IHC analysis, 5 µm tissue slides were added with a rabbit polyclonal antibody against IL6ST (1:100, diluted with 2% sheep serum, bs-34036R; Bioss, Boston, MA, USA) and incubated at 4°C overnight, followed by blocking with 5% sheep serum for 1 hour at 25°C. After that, the slides were washed with 0.01 mol/L phosphate buffer saline with Tween 20 (PBST), and then a two-step kit detection system (PV9000, Golden Bridge International, Beijing, China) was used according to the manufacturer’s instructions. The sections were stained with DAB (ZLI-9018, Golden Bridge International, Beijing, China) for 5-10 minutes at room temperature, and then counterstained with hematoxylin for 2 minutes. Finally, the sections were then scanned using a scanning holographic microscope (3DHISTECH; Pannoramic MIDI). The staining results were assigned an immunoreactive scoring system (IRS) score,^29^ considering both the staining intensity and the percentage of tumor cells with positive reactions. Each section was assessed by 2 individuals.

### Bioinformatic Analysis

In the The Cancer Genome Atlas (TCGA) database, 51 unpaired normal and 647 CRC samples (50 paired normal and CRC samples) from The Cancer Genome Atlas Colon Adenocarcinoma (TCGA-COAD) database were included for further analysis. The related RNA-seq data of IL6ST were extracted. Statistical analysis, receiver operating characteristic (ROC) curve, and immune infiltration were performed using R (v.3.6.3). ggplot2 (v.3.3.3) was used for plotting. pROC (v.1.17.0.1) was used for ROC analysis.

### Cell Lines and Cell Culture

The nonmalignant human colonic epithelial cell lines NCM-460, colon cancer cell lines HCT116, HT-29, SW620, and SW480 were purchased from Procell Life Science&Technology Co., Ltd. (Wuhan, China) and Shanghai Zhong Qiao Xin Zhou Biotechnology Co., Ltd. (Shanghai, China). NCM-460 and SW620 cell lines were grown in DMEM (Gibco, New York) containing 10% fetal bovine serum (FBS) (Gibco, New York) and 1% penicillin–streptomycin (Gibco). HCT116 and HT-29 cell lines were cultured in McCoy’s 5A medium (Gibco, New York) supplemented with 10% FBS (Gibco, New York) and 1% penicillin–streptomycin (Gibco, New York). SW480 cell lines were cultured in RPMI 1640 (Gibco, New York) with 10% FBS (Gibco, New York) and 1% penicillin–streptomycin (Gibco, New York). The cell viability, mitochondrial morphology, intracellular iron content, and ROS concentration of these cell lines will be determined.

### Transmission Electron Microscopy

We collected the cell deposit by centrifugation, removed the medium, and added the electron microscope fixative. The mixture was then resuspended and mixed at 4°C for 2-4 hours. Afterward, sample was centrifuged, discarded the supernatant, and added 0.1 M phosphate buffer (PB) (pH 7.4), which was mixed and rinsed for 3 minutes. Finally, we centrifuged again and added the cells to 1% agarose solution in 0.1 M PB (pH 7.4) prepared with 1% osmium tetroxide and fixed at room temperature for 2 hours away from tlight. Cells were dehydrated in different gradients of alcohol for 20 minutes each and in acetone for 15 minutes twice, followed by acetone osmotic embedding. The embedded plates were polymerized at 60°C for 48 hours in an oven. The blocks were sliced at 60–80 nm, and sliced by copper mesh, then stained with 2% uranyl acetate saturated alcohol solution for 8 minutes. They were washed with 70% alcohol for 3 times, washed with ultrapure water for 3 times, stained with 2.6% lead citrate solution for 8 minutes, washed with ultrapure water for 3 times, and then dried with filter paper. The slices of copper mesh were put into the copper mesh box and dried at room temperature overnight. The morphology of mitochondria was observed using transmission electron microscopy.

### RNA Isolation and Quantitative PCR Analysis

Total RNA from the cell lines and colon samples was extracted using TRIzol reagent (Lifetech, cat. no. 15596026). Complementary DNA (cDNA) was prepared using a FastKing RT Kit (with gDNase) (TIANGEN, China). The primers used for IL6ST were as follows: F : 5’-ACTGTTGATTATTCTACTGTGTA-3’ and R : 5’- AATTATGTGGCGGATTGG-3’. The expression of the target gene mRNA was verified and analyzed using GAPDH as an internal standard. The primers for GAPDH were as follows: forward, 5’-TTGCCCTCAACGACCACTTT-3’; reverse, 5’-TGGTCCAGGGGTCTTACTCC-3’. Quantitative PCR was performed as published.^30^ The relative transcript levels were calculated using the 2^−^^ΔΔCT^ method. All experiments were performed in triplicate.

### Establishment of Transfected Cell Lines

IL6ST shRNA was designed and synthesized by Genomeditech Co., Ltd. (Shanghai, China), and the shRNA targeted sequences used in this study were as follows: #1: GCAAGTGGGATCACCTATGAA, #2: GGACCAACTTCCTGTTGATGT, #3: CGGCCAGAAGATCTACAATTA. The vector used was pGMLV-SC5 RNAi. Full-length IL6ST cDNA was synthesized and cloned into the expression vector PGMLV-CMV-MCS-3×Flag-EF1-ZsGreen1-T2A-Puro using the forward primer 5’-GCGAATTCGAAGTATACCTCGAGGCCACCATGTTGACGTTGCAGACTTGGC-3’ and reverse primer 5’-GTCATGGTCTTTGTAGTCGGATCCCTGAGGCATGTAGCCGCCTT-3’. Stable cell lines overexpressing IL6ST were picked using 2 µg/mL puromycin and 10 µg/mL blasticidin. Quantitative PCR and western blotting were used to detect the mRNA expression levels of IL6ST after transfection.

### Western Blot Assay

RIPA buffer (TIANGEN, China) with 30 μL protease inhibitor (Roche) was used to lyse the cultured cells. The protein concentration was quantified using the BCA protein assay (TIANGEN, China). Total protein was separated by sodium dodecyl sulfate-polyacrylamide gel electrophoresis, transferred to a PVDF membrane (Millipore), and blocked with 5% bovine serum albumin for 1 hour at room temperature. The membranes were incubated overnight with primary antibodies at 4°C, further incubated with the corresponding secondary antibodies with shaking, and developed using ECL solutions (Applygen, China). The primary antibodies used in the current study included PTGS2 (Bioss bs-0732R; 1:1000), NOX1 (Bioss bs-3682R; 1:1000), FTH1 (Abcam ab75972, 1:500), GPX4 (Bioss bs-3884R; 1:500), ACSL4 (Abcam ab155282; 1:500), GAPDH (abmart P30008; 1:1000), and the secondary antibody anti-rabbit (CST 7074).

### CCK8 Analysis

The cell counting kit-8 was performed to investigate cell viability. Briefly, the cells were seeded in 96-well plates at a density of 5 × 10^3^ cells/well. A 10 μL CCK-8 solution was added to each well. After 24 hours, the color strength was determined by measuring the color intensity at 450 nm using an enzyme marker.

### Iron Assay

The total iron content in CRC cells was determined using an Iron Analysis Kit (ab83366, Abcam, UK). Briefly, 1 × 10^6^ cells were lysed in 100 μL of iron analysis buffer, and the insoluble material was removed by centrifugation at 16 000×*g* for 10 minutes at 4°C. Fifty μL samples were added to a 96-well plate, and the volume was brought to 100 μL with 50 μL of assay buffer. Then, 5 μL of iron assay buffer was added. Following 30 minutes of incubation at room temperature, each well was spiked with 100 μL of the iron probe and incubated in the dark for 60 minutes at room temperature. Finally, the absorptivity was calculated at 550 nm, and the iron concentrations were calculated based on the standard curve.

### Quantification of ROS Assay

To detect ROS, 10 μM H2DCFDA and DHR123 in ROS assay Kit (S0033S, Beyotime, China) were incubated with CRC cells at 37°C for 30 minutes. Subsequently, the cells were washed thrice with PBS and analyzed using flow cytometry.

### Statistical Analysis

Data were expressed as mean ± standard deviation. Bioinformatic analyses were performed using R (v.3.6.3). Mann–Whitney or two-way ANOVA tests were used to compare means between 2 or 3 groups. Spearman’s test was used to evaluate the association between IL6ST level and immune cell infiltration or ferroptosis-related genes. Experimental data were plotted and analyzed with GraphPad Prism (version 8.2.1). A *P*-value <.05 was considered statistically significant.

## Results

### IL6ST was Upregulated in CRC Tissues and Cells

**Figure 1.**
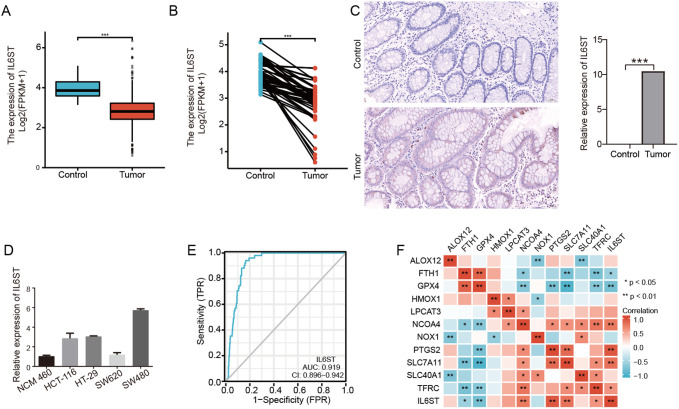
Upregulated IL6ST in CRC tissues and cells was associated with ferroptosis-related genes. (A) IL6ST expression is upregulated in CRC tissues compared with unpaired controls based on TCGA-COAD database (*P* = .000). (B) IL6ST expression is upregulated in CRC tissues compared with paired adjacent controls based on TCGA-COAD database (*P* = .000). (C) Higher expression of IL6ST in CRC tissues is found compared to adjacent controls via IHC assay (*P* = .0001) (200×). (D) IL6ST is upregulated in SW480, HCT116, HT-29, and SW620 cells than nonmalignant human colonic epithelial cell lines NCM-460. (E) Demonstrated the specificity and sensitivity of IL6ST in predicting CRC. (F) The expression level of IL6ST has a significant association with FTH1, GPX4, NCOA4, PTGS2, SLC7A11, and TFRC expression levels.

IL6ST expression was investigated in both unpaired and paired CRC tissues from TCGA-COAD database, and the expression of IL6ST was upregulated in CRC tissues compared to adjacent controls (unpaired *P* = .000, Figure 1A and paired *P* = .000, Figure 1B). Furthermore, we verified the expression of IL6ST in our CRC cohort and found higher expression of IL6ST in CRC tissues than in adjacent controls via IHC assay (*P* = .0001) (200×) (Figure 1C). The expression levels of IL6ST mRNA in various CRC cell lines were also measured. IL6ST was more highly expressed in SW480, HCT116, HT-29, and SW620 cells than in the nonmalignant human colonic epithelial cell line NCM-460 (Figure 1D). SW480 with the highest IL6ST expression was selected for further construction of knockdown cell-lines, and SW620 with the lowest IL6ST expression for the construction of overexpression stable cell lines. We then investigated the specificity and sensitivity of IL6ST in predicting CRC and found that the specificity and sensitivity of IL6ST in predicting CRC were high (AUC: 0.919, CI: 0.896-0.942) (Figure 1E). Furthermore, we explored the relationship between IL6ST and ferroptosis-related genes in CRC and found that IL6ST was significantly related to FTH1 (*r* = −0.116, *P* = .003), GPX4 (*r* = −0.369, *P* = 0.000), NCOA4 (*r* = 0.557, *P* = 0.000), PTGS2 (*r* = 0.458, *P* = .000), SLC7A11 (*r* = 0.434, *P* = .000), and TFRC (*r* = 0.201, *P* = .256E−07) (Figure 1F).

### IL6ST Inhibited Ferroptosis in SW480 and SW620 Cells

**Figure 2.**
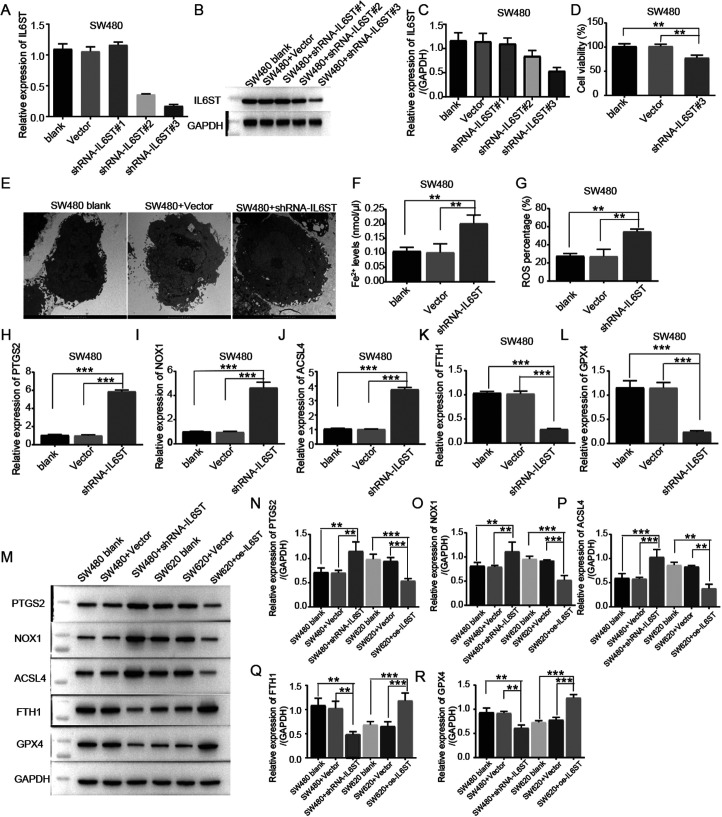
Knockdown of IL6ST could decrease viability and increase ferroptosis phenotype and related genes. (A-C) Effective knockdown of IL6ST in SW480 CRC cells is detected using RT-PCR. (B, C) Effective knockdown of IL6ST in SW480 CRC cells is detected using western blot. (D) Knockdown of IL6ST could significantly decrease the viability of SW480 CRC cells compared to nonmalignant human colonic epithelial cell lines NCM-460 and vector controls via CCK-8 assays (*P* = .007 and *P* = .004, respectively). (E) IL6ST knockdown SW480 cells exhibit shrunken and damaged mitochondria (3200×). (F) Knockdown of IL6ST up-regulated the levels of iron in SW480 cells compared to the control group (*P* = .007). (G) Knockdown of IL6ST increased the levels of ROS in SW480 cells compared to the control group (*P* = .005). (H-L) The expressions of PTGS2, NOX1, and ACSL4 are upregulated at the RNA levels, while FTH1 and GPX4 are downregulated in IL6ST knockdown SW480 cells (*P* < .01). (M-R) The expressions of PTGS2, NOX1, and ACSL4 are upregulated at protein levels in IL6ST knockdown SW480 cells, while FTH1 and GPX4 are upregulated in IL6ST overexpression SW620 cells (*P* < .01).

To evaluate the function of IL6ST in CRC ferroptosis, IL6ST-knockdown SW480 cells were generated. Then, IL6ST effective knockdown was detected in SW480 CRC cells (Figure 2A-C). CCK-8 assays revealed that IL6ST knockdown could significantly decrease the viability of SW480 CRC cells compared to that of the normal human colon epithelial cell line NCM-460 and vector controls (*P* = .007 and *P* = .004, respectively) (Figure 2D). To confirm the existence of ferroptosis in CRC cell lines, morphological changes were observed by electron microscopy. IL6ST-depleted SW480 cells exhibited shrunken and damaged mitochondria (3200×) (Figure 2E). Ferroptosis is triggered by iron-dependent accumulation of ROS; thus, intracellular ROS levels and iron accumulation were analyzed. Our findings revealed that IL6ST knockdown significantly increased the levels of iron and ROS in SW480 cells compared to those in the control group (*P* = .007 and *P* = .005, respectively) (Figure 2F and 2G). Furthermore, the expression levels of ferroptosis-related genes were investigated. Resulting profiles demonstrated that PTGS2, NOX1, and ACSL4 expression were upregulated at both the RNA and protein levels, while FTH1 and GPX4 were downregulated (*P* < .01) (Figure 2H-R).

**Figure 3.**
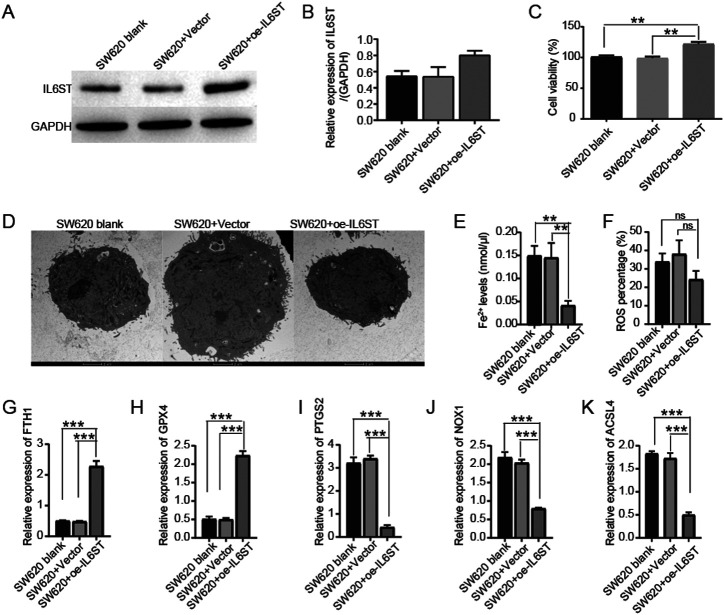
IL6ST overexpression increased cell viability and decreased ferroptosis phenotype and related genes. (A,B) Effective overexpression of IL6ST in SW620 CRC cells is detected using western blot. (C) Overexpression of IL6ST increased the viability of SW620 CRC cells via CCK-8 assays compared to NCM-460 or vector control. (D) IL6ST overexpression protected mitochondrial integrity (3200×). (E) IL6ST overexpression decreased iron accumulation. (F) IL6ST overexpression down-regulated the production of ROS. (G-K) IL6ST up-regulated the expression of FTH1 and GPX4 and decreased expression of PTGS2, NOX1, and ACSL4 at RNA levels (*P* < .01).

Furthermore, IL6ST overexpression cells in SW620 cells were generated. The effective overexpression of IL6ST in SW620 CRC cells was detected by western blotting (Figure 3A and 3B). To investigate the functional consequences of IL6ST overexpression, CCK-8 assays were conducted; overexpression of IL6ST was found to significantly increase the viability of SW620 CRC cells compared to the normal human colon epithelial cell line NCM-460 and vector control cells (*P* = .001, *P* = .001, respectively) (Figure 3C). IL6ST overexpression protected mitochondrial integrity (3200×) (Figure 3D) and decreased iron accumulation and ROS production compared to those in vector control cells (*P* = .006 and *P* = .05, respectively) (Figure 3E and 3F). IL6ST could promote the expression of FTH1 and GPX4 and reduced the expression of PTGS2, NOX1, and ACSL4 at the protein and RNA levels (*P* < .01) (Figures 2M-R and 3G-K).

### Correlation between IL6ST and Immune Cell Subtype Infiltration

**Figure 4.**
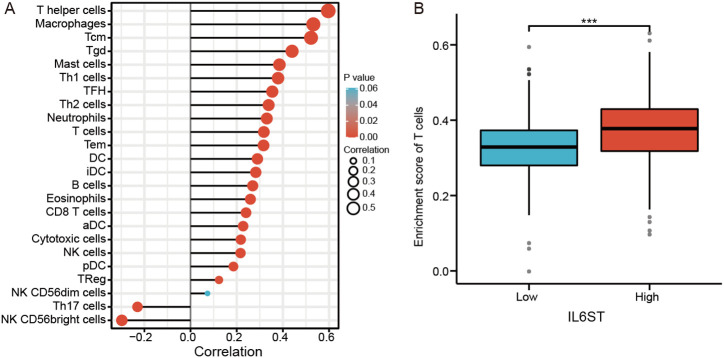
Correlation between IL6ST and immune cell subtype infiltration. (A) The expression level of IL6ST is positively correlated with the infiltration of T helper cells, macrophages, Tcm cells, Tgd cells, Mast cells, DC cells, Th1 cells, TFH cells, Th2 cells, neutrophils and T cells (*P* < .05). (B) The enrichment score of T cells is significantly higher in IL6ST high-expressed group than in the low-expressed group.

As IL6ST is involved in immune response, the correlation between IL6ST and immune cell infiltration was also examined. IL6ST expression was positively associated with the infiltration of helper T cells, macrophages, Tcm cells, Tgd cells, mast cells, DC, Th1 cells, TFH cells, Th2 cells, neutrophils, and T cells (*P* < .05; Figure 4A). The enrichment score of T cells was significantly higher in the high-expression group of IL6ST compared to the lower-expression group (Figure 4B).

## Discussion

IL6ST acts as a co-receptor for some cytokines and plays an important role in inflammation-associated CRC. Whether it affects ferroptosis is unknown, and the current study provides preliminary theoretical value for improving the efficacy of CRC by targeting IL6ST combined with ferroptosis inducers. The present study demonstrated that IL6ST was highly expressed in colon cancer tissues and could inhibit ferroptosis.

Ferroptosis is involved in both inflammation and cancer, indicating that it might be a bridge linking intestinal inflammation and CRC in developing CRC. Considering the role of ferroptosis inducers in the treatment of chemotherapy-resistant CRC, treatment combined with targets of inflammation-associated CRC could improve the efficiency of ferroptosis inducers in treating CRC. Our findings revealed that IL6ST was significantly highly expressed in CRC tissues than in the adjacent controls. This is supported by a study showing that IL6ST was activated in colitis-associated CRC and inhibited colon cancer by blocking IL6 signaling.^31^ By manipulating IL6ST, we confirmed that IL6ST could inhibit ferroptosis in CRC. Since the relationship between ferroptosis and colon cancer has been reported.^20,32^ and ferroptosis inducers have been used in clinical treatment,^33^ iron-inducer interventions were not performed to avoid resources wasting, which was a weakness of this study. In the future, we will add a ferroptosis-inducer intervention to provide more substantial evidence for the current results when we further investigate the mechanisms involved in IL6ST-induced ferroptosis. Furthermore, the correlation between IL6ST and immune cells was demonstrated by bioinformatics analysis. The current results are supported by previous studies that clarified the correlation between IL6ST and inflammatory diseases.^34^ As mentioned, knowledge about cross talk between ferroptosis and classic cell signaling pathways will point the way to future mechanistic studies.^35^ Inactivation of the AMPK pathway eliminates the protective effect of energy contingency against ferroptosis.^36^ In addition, cancer cells with high expression of AMPK activity in the basement membrane are resistant to ferroptosis, whereas cells with low AMPK activity were more sensitive to ferroptosis,^36^ indicating cross talk between ferroptosis and the AMPK signal pathway. PI3K–AKT–mTOR pathway is activated in most human cancers, and studies showed activation of this signaling could suppress ferroptosis through regulating sterol regulatory element-binding protein 1 (SREBP1)-mediated lipid metabolisms.^37^ Apart from that, the transcriptional regulatory activity of Yes-associated protein (YAP) could promote ferroptosis.^38^ IL6ST, as an upstream of PI3K–AKT–mTORC1 pathways, might inhibit ferroptosis through regulating PI3K–AKT–mTORC1 pathways, and further research is needed to prove this hypothesis.

However, there are some limitations in the present study. First, our number of clinical specimens is small, and the data are from a single center, which needs to be further validated by expanding the sample size in multiple centers. Second, we did not detect the number or proportion of immune cells in blood specimens and colon cancer tissue specimens, and further studies to detect immune cells in blood or tissues would be helpful in identifying which colon cancer patients respond to ferroptosis-related drugs. Third, we lacked validation in animal models or organoid tissues, and in vivo, animal experiments are needed in the future to further validate the relationship between IL6ST and ferroptosis. Fourth, this study investigated the correlation between IL6ST and ferroptosis without in-depth mechanistic studies, and we hope that in-depth mechanistic studies will be conducted in the future, which will provide a direction for the development of new drugs for the treatment of colorectal cancer. In the future, we hope clinical trials will be designed to evaluate the efficacy of ferroptosis inhibitors combined with IL6ST in CRC, and we look forward to the implementation of clinical translation.

Overall, we conclude that IL6ST may inhibit ferroptosis in colon cancer cells, providing a new combined target for enhancing the efficacy of ferroptosis inducers in the treatment of CRC.
